# Unfinished Business and Experiences of Bereaved Families of Patients with Cancer: A Cross-Sectional Survey

**DOI:** 10.1177/26892820261428901

**Published:** 2026-03-17

**Authors:** Masanori Mori, Tatsuya Morita, Kengo Imai, Toshihiro Yamauchi, Satoru Miwa, Soichiro Okamoto, Satoshi Inoue, Sayuri Itoga, Kaori Fukuta, Naosuke Yokomichi, Yumi Sakuma, Koichi Sugiyama, Keiko Tamura, Akemi Shirado Naito, Hiroyuki Otani, Mitsunori Miyashita

**Affiliations:** 1 Division of Palliative and Supportive Care, Seirei Mikatahara General Hospital, Hamamatsu, Japan.; 2 Seirei Mikatahara General Hospital, Seirei Hospice, Hamamatsu, Japan.; 3 Nursing, Seirei Mikatahara General Hospital, Hamamatsu, Japan.; 4 Osaka Dental University, Hirakata, Japan.; 5 Miyazaki Medical Association Hospital, Miyazaki, Japan.; 6 Department of Palliative Care Team, Palliative and Supportive Care, National Hospital Organization Kyushu Cancer Center, Fukuoka, Japan.; 7 Department of Palliative Care Team, Palliative and Supportive Care, St. Mary’s Hospital, Fukuoka, Japan.; 8 Department of Palliative Nursing, Tohoku University School of Medicine, Sendai, Japan.

**Keywords:** cancer, family, survey, unfinished business

## Abstract

**Background:**

Unfinished business (UB) is common among bereaved families of patients with advanced cancer and is associated with poor psychological outcomes, but remains underexplored.

**Objectives:**

To examine the prevalence, psychological impact, care-related factors, and families’ experiences associated with UB using multidimensional assessments.

**Design:**

A cross-sectional survey was conducted.

**Setting/Subjects:**

Bereaved family members of patients with cancer at a Japanese inpatient hospice.

**Measurements:**

UB was assessed using a one-item scale, the Unfinished Business in Bereavement Scale—Short (UBBS), and the Unfinished Business Scale—Family (UBS-F). Depression and complicated grief were measured using the Patient Health Questionnaire-2 (PHQ-2) and the Brief Grief Questionnaire (BGQ), respectively. Analyses included *t* tests, chi-square tests, and multivariate logistic regression.

**Results:**

Of 455 bereaved families, 319 (70%) participated. UB was reported by 53.9% (95% confidence interval [CI] = 48.4–59.4%) on the one-item scale and by 34.5–40.8% and 27.9–65.5% using UBBS and UBS-F, respectively. Using the one-item UB scale, families with UB had significantly higher respective depression (PHQ-2 = 1.73 vs. 1.13; *p* = 0.002) and grief (BGQ = 4.53 vs. 3.06; *p* < 0.001) scores. UB was more likely among families of younger patients (OR = 0.73 per 10-year increase), spouses (OR = 3.81), children (OR = 4.24), those less prepared for death (OR = 3.69–6.31), and those who felt clinicians were unconcerned about the family’s wishes (OR = 1.91). Many (71.8%) expressed a wish to do something for the patient but were preoccupied with daily responsibilities.

**Conclusions:**

UB is prevalent and associated with psychological distress among bereaved families of patients with cancer. Interventions promoting preparedness and supporting patients and families to realize wishes may reduce UB and improve bereavement outcomes.

## Key Message

In a survey of 319 bereaved families of patients with cancer, over half reported unfinished business, linked to higher levels of depression and grief. Risk factors included poor preparedness and limited support. The findings highlight the need for interventions that foster preparedness and help realize patient and family wishes before death.

## Introduction

One of the primary goals of palliative care is to support patients and families in achieving a “good death” with minimal distress, including that arising from unfinished business (UB).^1–3^ UB is defined as “incomplete, unexpressed, or unresolved relationship issues with the deceased”^4,5^ or as “a cognitive process that involves appraising one’s relationship with a deceased loved one as incomplete, unexpressed, or unresolved, lacking closure,”^4,6^ and it is associated with depression and complicated grief among bereaved families.^5,7^

In Western settings, the prevalence approaches 50%,^5^ and UB-related distress can exert greater psychological impact than UB itself.^5,6^ In Japan, a national survey reported lower prevalence (26%) based on a single-item measure, with most concerns involving unfulfilled wishes rather than conflict.^7^ Cultural values such as harmony and indirect communication may shape the nature and expression of UB in end-of-life (EOL) conversations.^8–11^ However, prior research has seldom explored bereaved families’ experiences or clinicians’ roles, and few studies have used multidimensional assessments or examined modifiable care factors.^5,6,12^ No interventional or quality improvement studies have addressed UB in advanced cancer care.

To address these gaps, we conducted a baseline survey at an inpatient hospice to examine UB prevalence using single- and multidimensional measures, explore bereaved families’ experiences, and identify psychological, clinical, and care-related correlates. Our goal was to provide foundational data for culturally appropriate interventions to support patients and families in addressing UB and alleviating psychological distress at EOL.

## Materials and Methods

### Design

This cross-sectional, anonymous questionnaire survey took place between October and November 2020.

### Participants

Eligible participants were bereaved family members of patients with cancer who died between April 2018 and March 2020 in the inpatient palliative care unit (PCU) at Seirei Mikatahara General Hospital, one of the designated cancer hospitals in Japan. Inclusion criteria were age ≥20 years for both patients and bereaved family members. Exclusion criteria included death due to treatment, inability to complete the questionnaire because of cognitive impairment or visual disability, or risk of serious psychological distress as determined by clinicians.

### Procedure

Questionnaires were mailed; returning a completed questionnaire implied consent. Participants were asked to return the completed questionnaires to the study secretariat office at Tohoku University in a different prefecture from that of the study site. A reminder was sent after one month. The ethical and scientific validity of the study was verified by the institutional review board of Seirei Mikatahara General Hospital (No. 20-31).

### Measurements

The questionnaire was developed based on a literature review and expert discussions.^4,7,12–15^ Face validity was confirmed by pilot testing with four people (one bereaved family member, one clinician, and two clinicians with bereavement experiences).

### UB scales

We provided an introductory statement: “People who have lost an important person in their life sometimes feel that something was left unsaid, unfinished, or unresolved in their relationship with the deceased,” and we utilized various scales to measure UBs.^5^ The primary endpoint was the one-item UB scale.

### One-item UB scale and UB-related distress

We asked participants: “Do you on the whole have any ‘unfinished business’ concerning the final few weeks you spent with the patient?” Participants were asked to respond on a 7-point Likert-type scale (1 = absolutely disagree, 2 = disagree, 3 = somewhat disagree, 4 = unsure, 5 = somewhat agree, 6 = agree, 7 = absolutely agree). We also asked them to indicate how distressed they had been about this issue in the past month. Participants were asked to respond on a 7-point Likert-type scale (1 = not distressed at all, 2 = not distressed, 3 = not very distressed, 4 = unsure, 5 = slightly distressed, 6 = distressed, 7 = extremely distressed). These scales were applied from previous studies on UB, and we modified response options to improve understanding.^1,5–7^

### Unfinished Business in Bereavement Scale (Short Version)

The short version of Unfinished Business in Bereavement Scale (UBBS), originally developed in the United States to assess UB in bereaved families, has two subscales: “Unfulfilled wishes” (four items) and “Unresolved conflict” (four items).^12^ The Japanese version of UBBS was created using the forward/backward translation method and used in our prior survey.^16^ Participants were asked to rate the level of distress associated with each UB item on a 7-point scale (from 1 = not at all distressed to 5 = extremely distressed), with higher scores indicating more UB. In the current study, we used the “Unfulfilled wishes” subscale because of its relevance to the PCU setting in Japan.^7^

### Unfinished Business Scale—Family

Unfinished Business Scale—Family (UBS-F) is a 10-item scale with three subscales of UB (Talk, Action, and Message) that we developed based on prior international and domestic studies regarding UB among bereaved families^5,7,12–14,17^ and discussion among the authors. Its validity and reliability have been confirmed in a separate study involving bereaved family members of patients with cancer in Japan.^16^ The *Talk* subscale includes four items (α = 0.91): “I wish I had talked about more things with the patient,” “I wish I had done more things with the assumption that he/she would die,” “I wish I had expressed the gratitude I had for the patient,” and “I wish I had listened more to the thoughts and real feelings of the patient.” The *Action* subscale includes three items (α = 0.98): “I wish I could have helped the patient fulfill what he/she wanted to do,” “I wish I could have helped the patient meet the person he/she wanted to see,” and “I wish I could have done something special for the patient.” Finally, the *Message* subscale includes three items (α = 0.87): “I wish I could have heard the gratitude and words of farewell from the patient,” “I wish the patient had left his/her thoughts, messages, and important things for me,” and “I wish I knew what the patient thought of me.” These items were graded on a 7-point Likert-type scale, from 1 = absolutely disagree to 7 = absolutely agree.

### Depression and grief

Depression was measured by the Patient Health Questionnaire 2 (PHQ-2), which is composed of two items used to assess the severity of depression.^18^ The validity and reliability of this scale have been confirmed in Japan.^19^ The score was then converted to a 0- to 6-point scale. A total score of 3 or greater was considered a valid cutoff point for depression.

Complicated grief was measured by the Brief Grief Questionnaire (BGQ), which is composed of five items, each rated on a scale from 0 to 2, with higher scores indicating more severe grief.^20^ The scores were then converted to a 0- to 10-point scale. A total score of 8 or greater indicated that the respondent was likely to develop complicated grief. Both the validity and reliability of this scale have been confirmed in Japan.^21^

### Care to relieve later UB

Based on the prior literature^7^ and discussions among the authors, we asked whether health care professionals provided care to reduce UB in the weeks before a patient’s death regarding three aspects: “Explanations provided by medical professionals when death was impending” (four items), “Bridges between the family’s and patient’s feelings” (two items), and “Measures to enable the family to do what they wanted before the patient’s death” (five items). Bereaved families responded either “yes” or “no.”

### Bereaved families’ experiences regarding UB

To obtain insights into bereaved families’ experiences regarding UB, we asked participants to answer questions concerning their experiences during the last few weeks they spent with their loved ones on a 4-point scale (1 = disagree/not applicable, 2 = somewhat agree, 3 = agree, 4 = absolutely agree). Responses of 1 and 2–4 were categorized as disagree and agree, respectively.

### Background

We also collected background data such as patients’ and families’ age, sex, and primary cancer site, as well as families’ relationship with the patient, education, household income, religion, physical and mental health during the patient’s last hospitalization, perceived social support, preparation for death, and history of psychiatric disorders. Preparation for death was measured on a 4-point Likert-type scale (“0 = not at all” to “3 = well-prepared”), with a higher score indicating greater preparation.^22^ To measure family-perceived social support from people around them, we used a brief, reliable, and widely used scale, designed to assess the content of support respondents perceived.^23,24^ The item was “degree of supportive listening,” and families responded based on a 5-point Likert-type scale (“0 = not at all” to “4 = a great deal”), with a higher score indicating greater perceived social support.

### Statistical analyses

We used descriptive statistics to summarize participants’ responses and calculated the mean (standard deviation [SD]) and rate of their responses with a 95% confidence interval (CI) as appropriate. For comparisons, responses regarding the one-item UB scale were divided into two categories: UB absent (1–4) and present (5–7). The rates of bereaved families with depression and complicated grief were compared between those with and without UB using chi-square tests. Depression and grief levels between those with and without UB were compared using *t* tests, and effect sizes were calculated using Cohen’s *d*. To explore the potential association between factors rated by families and the presence of UB, logistic univariate regression analyses were performed to screen baseline characteristics of patients and families, as well as care to relieve later UB as independent variables, and the presence of UB as a dependent variable. Finally, to identify independent determinants of having UB, all factors with a *p* value of <0.1 identified in univariate analyses were entered into multivariate logistic regression analysis.

For sample size calculation, we predicted that 25–40% of participants would have UB^7^ and determined that at least 128–164 subjects would be needed to calculate accuracy within a 15% width and 95% CI for a value of 25–40%. Considering missing data, responses from 200 participants were deemed sufficient.

In all statistical evaluations, *p* values of <0.05 were considered significant for the exploratory nature of the study. All analyses were performed using Statistical Package for the Social Sciences, version 28.0.

## Results

Questionnaires were sent to 455 bereaved families; 358 (79%) responded and 319 (70%) participated.

### Baseline characteristics

Families of younger patients, spouses or children, those with poorer mental status, and less prepared for death were more likely to report UB (Table 1).

**Table 1.** Background Characteristics of Patients and Bereaved Families

Variables	Total^a^ (*n* = 319)	UB, absent^a^ (*n* = 147)	UB, present^a^ (*n* = 172)	Odds ratio	95% CI	*p*
Patients						
Age, mean (SD)	73.64 (12.01)	75.69 (12.54)	71.88 (11.28)	0.97	0.95, 0.99	0.005
Age, per 10-year unit				0.80	0.66, 0.96	0.02
Sex						
Male	159 (49.8%)	79 (49.7%)	80 (50.3%)	Ref.		
Female	160 (50.2%)	68 (42.5%)	92 (57.5%)	1.34	0.86, 2.08	0.20
Primary cancer site						
Lung	79 (24.8%)	37 (46.8%)	42 (53.2%)	Ref.		
GI tract (esophagus, stomach, colon, rectum)	71 (22.3%)	36 (50.7%)	35 (49.3%)	0.86	0.45, 1.63	0.64
Gallbladder, bile duct, liver, pancreas	63 (19.7%)	30 (47.6%)	33 (52.4%)	0.97	0.50, 1.88	0.93
Breast	25 (7.8)	12 (48.0%)	13 (52.0%)	0.95	0.39, 2.35	0.92
Genitourinary (prostate, kidney, bladder)	18 (5.6%)	6 (33.3%)	12 (66.7%)	1.76	0.60, 5.16	0.30
Gynecological (uterus, ovary)	18 (5.6%)	7 (38.9%)	11 (61.1%)	1.38	0.49, 3.94	0.54
Blood (leukemia, lymphoma, myeloma)	5 (1.6%)	4 (80.0%)	1 (20.0%)	0.22	0.02, 2.06	0.19
Other (head and neck, soft tissue, brain, unknown primary, other)	40 (12.5%)	15 (37.5%)	25 (62.5%)	1.47	0.68, 3.20	0.33
Family						
Age, mean (SD)	62.90 (11.56)	63.03 (11.17)	62.78 (11.92)	1.00	0.98, 1.02	0.85
Age, per 10-year unit				0.93	0.77, 1.13	0.48
Sex						
Male	123 (38.6%)	58 (47.2%)	65 (52.8%)	Ref.		
Female	190 (59.6%)	87 (45.8%)	103 (54.2%)	1.06	0.67, 1.67	0.81
Relationship with the patient						
Spouse	133 (41.7%)	48 (36.1%)	85 (63.9%)	3.73	1.95, 7.15	<0.001
Child	118 (37.0%)	55 (46.6%)	63 (53.4%)	2.41	1.25, 4.64	0.01
Other	59 (18.5%)	40 (67.8%)	19 (32.2%)	Ref.		
Physical health status						
1. Good	95 (29.8%)	46 (48.4%)	49 (51.6%)	Ref.		
2. Fair	150 (47.0%)	69 (46.0%)	81 (54.0%)	1.10	0.66, 1.84	0.71
3. Poor	55 (17.2%)	22 (40.0%)	33 (60.0%)	1.41	0.72, 2.76	0.32
4. Very poor	12 (3.8%)	5 (41.7%)	7 (58.3%)	1.31	0.39, 4.44	0.66
Physical health status, mean (SD)	1.95 (0.80)	1.90 (0.78)	1.99 (0.81)	1.15	0.87, 1.52	0.34
Mental health status						
1. Good	39 (12.2%)	25 (64.1%)	14 (35.9%)	Ref.		
2. Fair	152 (47.6%)	66 (43.4%)	86 (56.6%)	2.33	1.12, 4.82	0.02
3. Poor	92 (28.8%)	41 (44.6%)	51 (55.4%)	2.22	1.03, 4.81	0.04
4. Very poor	27 (8.5%)	12 (44.4%)	15 (55.6%)	2.23	0.82, 6.08	0.12
Mental health status, mean (SD)	2.35 (0.81)	2.28 (0.85)	2.40 (0.77)	1.22	0.92, 1.61	0.17
Social support (degree of supportive listening)						
0. Not at all	3 (0.9%)	1 (33.3%)	2 (66.7%)	Ref.		
1. Little	13 (4.1%)	6 (46.2%)	7 (53.8%)	0.58	0.04, 8.15	0.69
2. Somewhat	81 (25.4%)	31 (38.3%)	50 (61.7%)	0.81	0.07, 9.27	0.86
3. Good	150 (47.0%)	66 (44.0%)	84 (56.0%)	0.64	0.06, 7.17	0.72
4. A great deal	62 (19.4%)	38 (61.3%)	24 (38.7%)	0.32	0.03, 3.68	0.36
Preparation for death						
0. Not at all	13 (4.1%)	3 (23.1%)	10 (76.9%)	6.57	1.70, 25.42	0.01
1. Very little	31 (9.7%)	9 (29.0%)	22 (71.0%)	4.82	2.01, 11.57	<0.001
2. Somewhat	167 (52.4%)	64 (38.3%)	103 (61.7%)	3.17	1.90, 5.30	<0.001
3. Well-prepared	104 (32.6%)	69 (66.3%)	35 (33.7%)	Ref.		
Religion						
No	121 (37.9%)	57 (47.1%)	64 (52.9%)	Ref.		
Yes	193 (60.5%)	87 (45.1%)	106 (54.9%)	1.09	0.69, 1.71	0.73
Education						
Elementary/junior high/high school	197 (61.7%)	89 (45.2%)	108 (54.8%)	Ref.		
University/graduate school/other	112 (35.1%)	54 (48.2%)	58 (51.8%)	0.89	0.56, 1.41	0.61
Household income						
<4 million yen (including unknown/prefer not to say)	219 (68.7%)	101 (46.1%)	118 (53.9%)	Ref.		
≥4 million yen	92 (28.8%)	41 (44.6%)	51 (55.4%)	1.07	0.65, 1.74	0.80
History of psychiatric illnesses						
No	274 (85.9%)	131 (47.8%)	143 (52.2%)	Ref.		
Yes	36 (11.3%)	13 (36.1%)	23 (63.9%)	1.62	0.79, 3.33	0.19

Total percentages do not equal 100% because of missing values.

a Values are means (SD) or *n* (%). Participants were categorized into UB absent and present groups that represent those with responses of 1–4 (“absolutely disagree” to “unsure”) and 5–7 (“somewhat agree” to “absolutely agree”) to the one-item unfinished business scale, respectively.

CI, confidence interval; GI, gastrointestinal; Ref., reference; SD, standard deviation; UB, unfinished business.

### Prevalence of UB using various tools

**FIG. 1.**
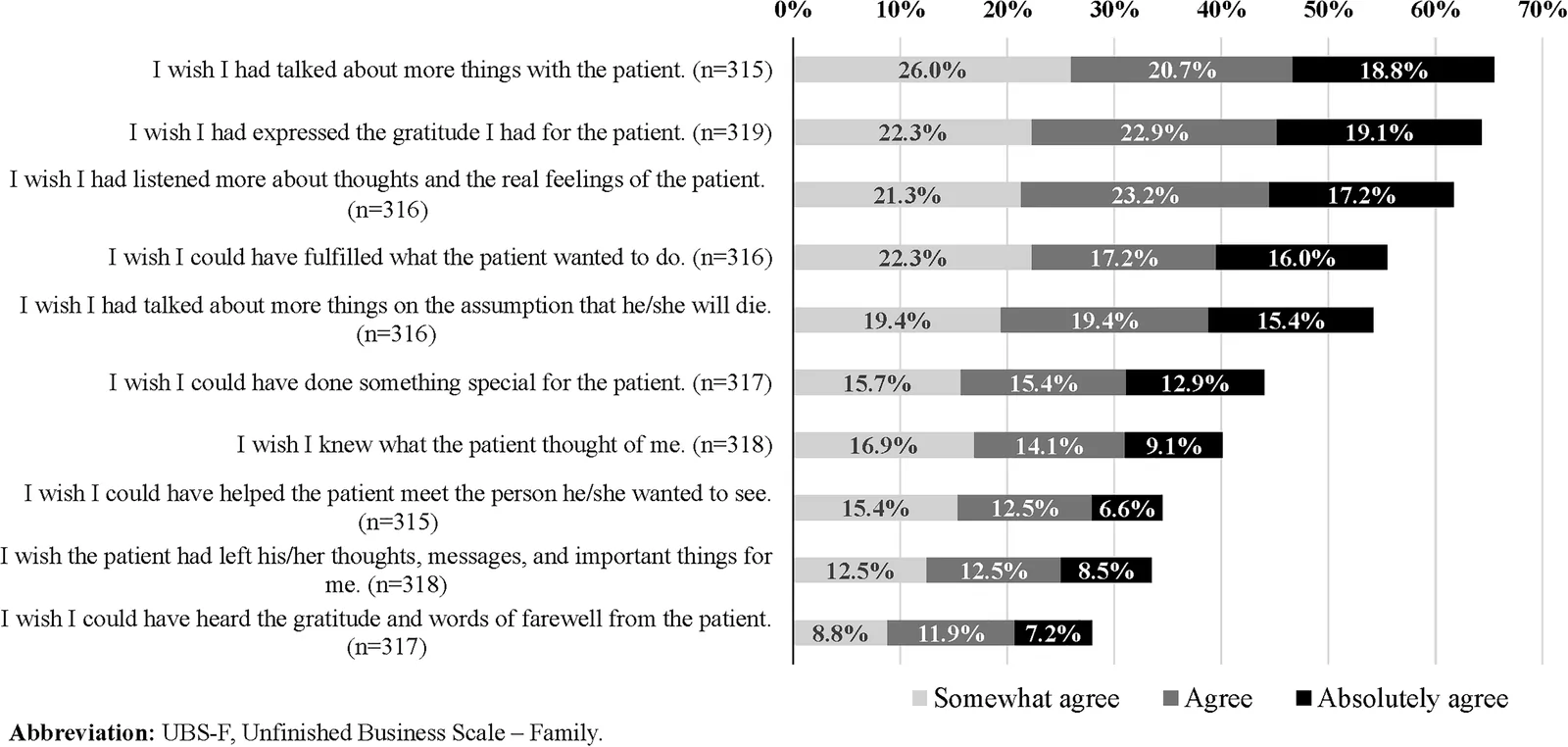
Rates of bereaved families reporting varying degrees of unfinished business (UBS-F). UBS-F, Unfinished Business Scale—Family.

Using the one-item UB scale, 172 (53.9%; 95% CI = 48.4–59.4%) had UB with varying degrees, and up to 191 (59.9%; 95% CI = 54.7–65.5%) families had various degrees of UB-related distress (Table 2). The short version of UBBS showed that 110 (34.5%; 95% CI = 30.0–40.6%) to 130 (40.8%; 95% CI = 35.9–46.8%) families felt distressed a lot or extremely distressed regarding the 4-item scales of unfulfilled wishes (Table 3). Using UBS-F, 173 (54.2%; 95% CI = 49.2–60.2%) to 209 (65.5%; 95% CI = 60.9–71.4%), 110 (34.5%; 95% CI = 29.8–40.4%) to 177 (55.5%; 95% CI = 50.4–61.4%), and 89 (27.9%; 95% CI = 23.4–33.3%) to 128 (40.1%; 95% CI = 35.0–45.8%) families had various degrees based on UB on Talk, Action, and Message subscales, respectively (Table 4, Fig. 1).

**Table 2.** Prevalence of Unfinished Business Assessed by Various Scales: One-Item Unfinished Business Scale and Unfinished Business-Related Distress

	Mean (SD)	Somewhat to absolutely agree (5–7) *n* (%)	1. Absolutely disagree	2. Disagree	3. Somewhat disagree	4. Unsure	5. Somewhat agree	6. Agree	7. Absolutely agree
Overall UB (*n* = 319)	4.4 (1.8)	172 (53.9%)	17 (5.3%)	61 (19.1%)	22 (6.9%)	47 (14.7%)	77 (24.1%)	54 (16.9%)	41 (12.9%)

	Mean (SD)	Slightly to extremely distressed (5–7) *n* (%)	1. Not distressed at all	2. Not distressed	3. Not very distressed	4. Unsure	5. Slightly distressed	6. Distressed	7. Extremely distressed
UB-related distress (*n* = 317)	4.7 (1.6)	191 (59.9%)	12 (3.8%)	25 (7.8%)	32 (10.0%)	57 (17.9%)	81 (25.4%)	63 (19.7%)	47 (14.7%)

UB, unfinished business; SD, standard deviation.

**Table 3.** Prevalence of Unfinished Business Assessed by the Unfinished Business in Bereavement Scale, Short Version

	Mean (SD)	Distressed a lot and extremely distressed (4–5) *n* (%)	1. Not at all distressed	2. A little distressed	3. Neutral	4. Distressed a lot	5. Extremely distressed
I wish we did more things together. (*n* = 315)	3.2 (1.2)	130 (40.8%)	19 (6.0%)	84 (26.3%)	82 (25.7%)	78 (24.5%)	52 (16.3%)
I should have told him/her ‘I love you’ more often. (*n* = 313)	3.1 (1.2)	117 (36.7%)	28 (8.8%)	75 (23.5%)	93 (29.2%)	76 (23.8%)	41 (12.9%)
I wish I had told _______ how much s/he meant to me. (*n* = 312)	3.1 (1.2)	118 (37.0%)	32 (10.0%)	61 (19.1%)	101 (31.7%)	73 (22.9%)	45 (14.1%)
I wish I would have taken my chance to say goodbye. (*n* = 313)	3.1 (1.2)	110 (34.5%)	35 (11.0%)	65 (20.4%)	103 (32.3%)	61 (19.1%)	49 (15.4%)
Mean (SD)	3.1 (1.0)						
Total score, mean (SD)	12.5 (4.2)						

SD, standard deviation.

**Table 4.** Prevalence of Unfinished Business Assessed by the Unfinished Business Scale—Family

	Mean (SD)	Somewhat to absolutely agree (5–7) *N* (%)	1. Absolutely disagree	2. Disagree	3. Somewhat disagree	4. Unsure	5. Somewhat agree	6. Agree	7. Absolutely agree
Talk subscale, mean (SD)	4.7 (1.6)								
I wish I had talked about more things with the patient. (*n* = 315)	4.8 (1.7)	209 (65.5%)	14 (4.4%)	32 (10.0%)	23 (7.2%)	37 (11.6%)	83 (26.0%)	66 (20.7%)	60 (18.8%)
I wish I had talked about more things on the assumption that he/she will die. (*n* = 316)	4.5 (1.9)	173 (54.2%)	23 (7.2%)	44 (13.8%)	21 (6.6%)	55 (17.2%)	62 (19.4%)	62 (19.4%)	49 (15.4%)
I wish I had expressed the gratitude I had for the patient. (*n* = 319)	4.8 (1.8)	205 (64.3%)	21 (6.6%)	27 (8.5%)	21 (6.6%)	45 (14.1%)	71 (22.3%)	73 (22.9%)	61 (19.1%)
I wish I had listened more about thoughts and the real feelings of the patient. (*n* = 316)	4.7 (1.8)	197 (61.8%)	18 (5.6%)	34 (10.7%)	24 (7.5%)	43 (13.5%)	68 (21.3%)	74 (23.2%)	55 (17.2%)
Action subscale, mean (SD)	4.2 (1.6)								
I wish I could have fulfilled what the patient wanted to do. (*n* = 316)	4.6 (1.8)	177 (55.5%)	20 (6.3%)	33 (10.3%)	28 (8.8%)	58 (18.2%)	71 (22.3%)	55 (17.2%)	51 (16.0%)
I wish I could have helped the patient meet the person he/she wanted to see. (*n* = 315)	3.9 (1.7)	110 (34.5%)	34 (10.7%)	52 (16.3%)	33 (10.3%)	86 (27.0%)	49 (15.4%)	40 (12.5%)	21 (6.6%)
I wish I could have done something special for the patient. (*n* = 317)	4.2 (1.8)	140 (43.9%)	23 (7.2%)	53 (16.6%)	29 (9.1%)	72 (22.6%)	50 (15.7%)	49 (15.4%)	41 (12.9%)
Message subscale, mean (SD)	3.7 (1.6)								
I wish I could have heard the gratitude and words of farewell from the patient. (*n* = 317)	3.5 (1.8)	89 (27.9%)	43 (13.5%)	88 (27.6%)	26 (8.2%)	71 (22.3%)	28 (8.8%)	38 (11.9%)	23 (7.2%)
I wish the patient had left his/her thoughts, messages, and important things for me. (*n* = 318)	3.8 (1.8)	107 (33.5%)	31 (9.7%)	75 (23.5%)	27 (8.5%)	78 (24.5%)	40 (12.5%)	40 (12.5%)	27 (8.5%)
I wish I knew what the patient thought of me. (*n* = 318)	3.9 (1.8)	128 (40.1%)	31 (9.7%)	72 (22.6%)	20 (6.3%)	67 (21.0%)	54 (16.9%)	45 (14.1%)	29 (9.1%)
Total score, mean (SD)	4.3 (1.4)								

SD, standard deviation.

### Association of UB and mental health

Bereaved families with UB tended to show a higher rate of depression (total score of PHQ-2 ≥ 3) than those without UB (22.2% [37/166] vs. 14.1% [20/142], respectively; *p* = 0.065). Families with UB showed a higher rate of complicated grief (total score of BGQ ≥ 8) than those without UB (13.1% [22/168] vs. 4.96% [7/141], respectively, *p* = 0.015). Also, there were significant differences in mean scores of PHQ-2 (1.73 [SD = 1.75] vs. 1.13 [1.63], respectively; effect size, 0.36; *p* = 0.002) and BGQ (4.53 [2.33] vs. 3.06 [2.34], respectively; effect size, 0.63; *p* < 0.001) based on the presence or absence of UB, respectively.

### Care to relieve later UB

Bereaved family members reported the presence or absence of various types of care given by health care providers (Table 5). Families of patients whose health care providers did not explain in detail how long the patient would be able to talk and move were more likely to report UB compared with those whose providers did offer such explanations (65.1% vs. 50.9%, respectively; odds ratio [OR] = 1.80; 95% CI = 1.07–3.03; *p* = 0.03). Similarly, families who felt that doctors and nurses were not concerned about what the family wanted to do before the patient’s death were more likely to report UB than those who felt that doctors and nurses were concerned (64.0% vs. 50.5%, respectively; OR = 1.75; 95% CI = 1.05–2.91; *p* = 0.03).

**Table 5.** The Prevalence of Care as Perceived by Bereaved Families With or Without Unfinished Business

Care	*n* (%)	UB, absent	UB, present	OR	95% CI	*p*
Explanations provided by medical professionals when death was impending						
They explained to help me concretely visualize future changes in the patient’s pathological condition. (*n* = 312)						
Yes	279 (87.5%)	134 (48.0%)	145 (52.0%)	Ref.		
No	33 (10.3%)	10 (30.3%)	23 (69.7%)	2.13	0.98, 4.63	0.05
They explained in detail how long the patient would be able to talk and move. (*n* = 305)						
Yes	222 (69.6%)	109 (49.1%)	113 (50.9%)	Ref.		
No	83 (26.0%)	29 (34.9%)	54 (65.1%)	1.80	1.07, 3.03	0.03
They explained that the patient’s life expectancy might be a few days. (*n* = 312)						
Yes	263 (82.4%)	127 (48.3%)	136 (51.7%)	Ref.		
No	49 (15.4%)	17 (34.7%)	32 (65.3%)	1.76	0.93, 3.32	0.08
They told me that even though the patient appeared to be unconscious, he/she could hear until the end. (*n* = 304)						
Yes	211 (66.1%)	99 (46.9%)	112 (53.1%)	Ref.		
No	93 (29.2%)	37 (39.8%)	56 (60.2%)	1.34	0.82, 2.20	0.25
Bridges between the family’s and patient’s feelings						
There were times when medical professionals conveyed the patient’s feelings to me (the family) instead. (*n* = 295)						
Yes	153 (48.0%)	72 (47.1%)	81 (52.9%)	Ref.		
No	142 (44.5%)	58 (40.8%)	84 (59.2%)	1.29	0.81, 2.04	0.28
There were times when medical professionals conveyed my (the family’s) feelings to the patient instead. (*n* = 291)						
Yes	134 (42.0%)	64 (47.8%)	70 (52.2%)	Ref.		
No	157 (49.2%)	66 (42.0%)	91 (58.0%)	1.26	0.79, 2.01	0.33
Measures to enable the family to do what they wanted before the patient’s death						
The doctors and nurses were concerned about what the family wanted to do before the patient’s death. (*n* = 305)						
Yes	216 (67.7%)	107 (49.5%)	109 (50.5%)	Ref.		
No	89 (27.9%)	32 (36.0%)	57 (64.0%)	1.75	1.05, 2.91	0.03
The doctors and nurses gave specific suggestions on how the family could do what they wanted for the patient (such as taking a walk with the patient in bed). (*n* = 297)						
Yes	183 (57.4%)	83 (45.4%)	100 (54.6%)	Ref.		
No	114 (35.7%)	49 (43.0%)	65 (57.0%)	1.10	0.69, 1.76	0.69
They gave clear instructions on when to do the things the family wanted to do (such as saying: “Now is the best time to go out with the patient”). (*n* = 289)						
Yes	153 (48.0%)	73 (47.7%)	80 (52.3%)	Ref.		
No	136 (42.6%)	56 (41.2%)	80 (58.8%)	1.30	0.82, 2.08	0.27
The doctors and nurses helped the family create good memories with the patient (such as taking photos and holding events). (*n* = 300)						
Yes	164 (51.4%)	78 (47.6%)	86 (52.4%)	Ref.		
No	136 (42.6%)	57 (41.9%)	79 (58.1%)	1.26	0.80, 1.99	0.33
They thought about what was feasible together until the end of the patient’s life so that the family could do what they wanted to in the time they had. (*n* = 296)						
Yes	179 (56.1%)	85 (47.5%)	94 (52.5%)	Ref.		
No	117 (36.7%)	49 (41.9%)	68 (58.1%)	1.26	0.78, 2.01	0.34

OR, odds ratio.

The absence or presence of unfinished business was categorized using the one-item unfinished business scale, with responses scored from 1 to 4 (Absolutely disagree to Unsure) versus 5 to 7 (Somewhat to Absolutely agree), respectively.

### Factors associated with UB

Multivariate analysis revealed that bereaved families of younger patients (OR = 0.73 per 10-year increase; 95% CI = 0.57–0.94; *p* = 0.01), family members who were spouses (OR = 3.81; 95% CI = 1.83–7.94; *p* < 0.001) or children (OR = 4.24; 95% CI = 1.90–9.43; *p* < 0.001), those who felt less prepared for the patient’s death (“not at all”: OR = 5.00; 95% CI = 1.14–21.90; *p* = 0.03; “very little”: OR = 6.31; 95% CI = 2.16–18.43; *p* < 0.001; “somewhat”: OR = 3.69; 95% CI = 2.10–6.49; *p* < 0.01, compared with “well-prepared”), and those who reported that the doctors and nurses were not concerned about what the family wanted to do before the patient’s death (OR = 1.91; 95% CI = 1.05–3.45; *p* = 0.03) were significantly and independently more likely to report UB (Table 6).

**Table 6.** Factors Associated with Unfinished Business: Multivariate Analysis

Variables	OR	95% CI	*p*
Patient age, per 10-year increase	0.73	0.57, 0.94	0.01
Relationship with the patient			
Spouse	3.81	1.83, 7.94	<0.001
Child	4.24	1.90, 9.43	<0.001
Other	Ref.		
Preparation for death			
0. Not at all	5.00	1.14, 21.90	0.03
1. Very little	6.31	2.16, 18.43	<0.001
2. Somewhat	3.69	2.10, 6.49	<0.001
3. Well-prepared	Ref.		
The doctors and nurses were concerned about what the family wanted to do before the patient’s death.			
Yes	Ref.		
No	1.91	1.05, 3.45	0.03

The absence or presence of unfinished business was categorized using the one-item unfinished business scale, with responses scored from 1 to 4 (Absolutely disagree to Unsure) versus 5 to 7 (Somewhat to Absolutely agree), respectively.

Variables with *p* < 0.1 in patient and family characteristics (Table 1) and care perceived by bereaved families (Table 5) were used for multivariate analysis.

### Experiences of bereaved family members

With respect to explanations provided by the doctors and nurses, more than half of the respondents (56.4%) agreed with the statement: “Looking back, even if I had been told earlier that the time left was short, I think I would have accepted it” (Table 7). At the same time, 32% of bereaved families agreed with the statement: “Looking back, I wish they had told me earlier that the time left was short, but I think I might not have accepted it at the time.” Regarding their own actions, the majority of the respondents (71.8%) agreed: “I wanted to do something for the patient, but I was fully occupied with managing my daily life.” As for conversations with the patient, approximately half of the respondents (50.2%) indicated: “I avoided conversations on death with the patient, but I wish we could have talked about it more frankly.” Finally, concerning regrets, the majority (87.8%) stated: “I have some regrets, but if I ask myself what more I could have done at that time, I think I was doing my best then.”

**Table 7.** Bereaved Families’ Experiences

Experiences	*n* (%)	UB, absent	UB, present
About explanations provided by the doctors and nurses			
Many of their explanations were based on the premise that the patient was going to die, and sometimes I felt scared			
Disagree	273 (85.6%)	131 (48.0%)	142 (52.0%)
Agree	38 (11.9%)	11 (28.9%)	27 (71.1%)
Missing	8 (2.5%)		
They explained on the premise that the patient was going to die even in front of him/her, and I thought they were insensitive			
Disagree	291 (91.2%)	135 (46.4%)	156 (53.6%)
Agree	18 (5.6%)	6 (33.3%)	12 (66.7%)
Missing	10 (3.1%)		
They asked if I had any regrets, but I thought it was none of their business			
Disagree	294 (92.2%)	135 (45.9%)	159 (54.1%)
Agree	9 (2.8%)	2 (22.2%)	7 (77.8%)
Missing	16 (5.0%)		
They explained that time was limited (death was impending), but I did not know how to treat the patient			
Disagree	194 (60.8%)	104 (53.6%)	90 (46.4%)
Agree	114 (35.7%)	36 (31.6%)	78 (68.4%)
Missing	11 (3.4%)		
Looking back, I wish they had told me earlier that the time left was short, but I think I might not have accepted it at the time			
Disagree	205 (64.3%)	115 (56.1%)	90 (43.9%)
Agree	102 (32.0%)	25 (24.5%)	77 (75.5%)
Missing	12 (3.8%)		
Looking back, even if I had been told earlier that the time left was short, I think I would have accepted it			
Disagree	121 (37.9%)	60 (49.6%)	61 (50.4%)
Agree	180 (56.4%)	79 (43.9%)	101 (56.1%)
Missing	18 (5.6%)		
About what families did			
I wanted to do something for the patient, but I was fully occupied with managing my daily life			
Disagree	78 (24.5%)	54 (69.2%)	24 (30.8%)
Agree	229 (71.8%)	86 (37.6%)	143 (62.4%)
Missing	12 (3.8%)		
I wish medical professionals had told me more clearly what I (the family) should do now			
Disagree	203 (63.6%)	105 (51.7%)	98 (48.3%)
Agree	101 (31.7%)	33 (32.7%)	68 (67.3%)
Missing	15 (4.7%)		
About conversations with the patient			
I wanted to ask the patient what he/she thought, but I was too afraid to ask			
Disagree	159 (49.8%)	94 (59.1%)	65 (40.9%)
Agree	149 (46.7%)	47 (31.5%)	102 (68.5%)
Missing	11 (3.4%)		
I avoided conversations on death with the patient, but I wish we could have talked about it more frankly			
Disagree	149 (46.7%)	92 (61.7%)	57 (38.3%)
Agree	160 (50.2%)	50 (31.3%)	110 (68.8%)
Missing	10 (3.1%)		
If medical professionals knew any thoughts of the patient, I wish they had conveyed them to the family more			
Disagree	183 (57.4%)	90 (49.2%)	93 (50.8%)
Agree	126 (39.5%)	52 (41.3%)	74 (58.7%)
Missing	10 (3.1%)		
*About regrets*			
I have some regrets, but if I ask myself what more I could have done at that time, I think I was doing my best then			
Disagree	32 (10.0%)	28 (87.5%)	4 (12.5%)
Agree	280 (87.8%)	114 (40.7%)	166 (59.3%)
Missing	7 (2.2%)		
I think my regrets might have been reduced more, depending on the method of support from medical professionals			
Disagree	212 (66.5%)	110 (51.9%)	102 (48.1%)
Agree	79 (24.8%)	24 (30.4%)	55 (69.6%)
Missing	28 (8.8%)		

The absence or presence of unfinished business was categorized using the one-item unfinished business scale, with responses scored from 1 to 4 (Completely disagree to Unsure) versus 5 to 7 (Somewhat to Completely agree), respectively.

Responses to experience items were categorized into two groups: No (“Disagree/Not applicable”) and Yes (“Somewhat agree to Absolutely agree” [scores 2–4]).

## Discussion

### Main findings

First, we found a high prevalence of UB among bereaved families of patients with cancer in Japan, with over half reporting UB and nearly 60% UB-related distress. Multidimensional scales revealed nuanced domains, with the Talk subscale most endorsed, followed by the Action subscale—suggesting regrets about missed conversations or actions rather than unreceived messages. These findings align with cultural norms that may limit direct EOL communication.^14,25^ Prevalence exceeded prior Japanese reports, likely reflecting finer-grained, multidimensional tools^7^; simplified measures may underestimate the complexity of UB. Importantly, UB was significantly correlated with depression and complicated grief, consistent with prior studies.^5,7^ Although causality cannot be inferred, addressing UB during patients’ final days may improve bereavement outcomes.

Second, several factors were associated with UB: being a spouse or child, lower preparedness for death, and perceived inadequate clinician concern. Poor preparedness was the strongest predictor, indicating that enhancing emotional, cognitive, and practical readiness may reduce UB.^7^ Although many families engage in EOL discussions,^11,26^ these must translate into meaningful preparation and closure.^27^

Third, families often wanted earlier prognostic disclosure yet they might not have accepted it at the time, reflecting diverse preferences and emotional readiness. Families also cited being overwhelmed by daily responsibilities, which limited their ability to act in ways they later valued. A recent Taiwanese study showed that the overall aim of families’ preparations and actions before their loved one’s death is getting everything right to have no regrets between the dead and living.^28^ Among the main themes was fulfilling caregiving responsibilities to minimize their loved one’s regret, even if it involved sacrifices in daily living.^28^ These findings illustrate the emotional and practical complexity of EOL caregiving and underscore the need for individualized, flexible approaches that respect differing capacities and wishes.

### Clinical and research implications

UB is a subtle but significant distress. Clinicians should remain attentive to the risk of UB, particularly among families of younger patients or those exhibiting signs of poor preparedness. Proactive strategies may include sensitive prognostic communication, encouragement of legacy work or meaningful conversations, and practical support that enables families to focus on what matters most in the final days. In addition, fostering open communication, incorporating preparatory discussions into daily care, and inviting families to consider meaningful actions may help mitigate UB. Providers should also be mindful of the ambivalence families may experience, wanting to know yet hesitating to receive prognostic information, as well as their varying readiness to address UB amid competing responsibilities in daily life.

Future research should pursue a multiphase agenda. First, observational studies should document real-world practices in prognostic communication and UB-related care in palliative care. Second, qualitative research involving clinicians may help identify nuanced practices that alleviate UB. Third, a multidimensional UB scale for terminally ill patients themselves should be developed to complement family perspectives. Finally, interventional studies are needed to evaluate culturally appropriate and individualized strategies, such as person-centered and family-based discussions or structured prompts for wishes, to reduce UB.^9,29^ Nationwide surveys comparing sites with and without quality improvement initiatives would also help assess the impact.

### Strengths and limitations

The strengths of this study were the use of both newly validated and internationally adapted UB measures and the in-depth exploration of bereaved families’ experiences. However, several limitations should be noted. First, this was a single-site study in Japan focusing on bereaved families of patients with cancer, and so generalizability outside of an inpatient hospice or Japan may be limited. To partially overcome this, we are currently conducting a nationwide survey.^30^ Second, recall bias may have been introduced because the survey was conducted approximately 6–30 months after each patient’s death. However, the high prevalence of UB despite the length of time between the death and survey administration suggests that the feelings and distress of UB may not easily diminish over time. Third, the content of the care and experience questionnaire was not conceptualized or validated. Therefore, our findings should be considered exploratory.

## Conclusions

UB is highly prevalent among bereaved families of patients with advanced cancer in Japan and is associated with poorer psychological outcomes. Addressing communication, preparedness, and meaningful engagement may reduce UB and improve EOL experiences. Future studies should develop and evaluate culturally appropriate and individualized interventions to reduce UB and enhance EOL experiences for both patients and families.

## Authors’ Contributions

All authors contributed to the study conception and design. Material preparation, data collection, and analysis were conducted by all authors. The first draft of the article was written by M.M., and all authors commented on previous versions of the article. All authors read and approved the final article.

## Data Availability Statement

The data of this study are available from Masanori Mori upon reasonable request.
